# Risk Stratification Using a Perioperative Nomogram for Predicting the Mortality of Bladder Cancer Patients Undergoing Radical Cystectomy

**DOI:** 10.3390/jcm14165810

**Published:** 2025-08-16

**Authors:** Daniel-Vasile Dulf, Anamaria Larisa Burnar, Patricia-Lorena Dulf, Doina-Ramona Matei, Hendea Raluca Maria, Cătălina Bungărdean, Maximilian Buzoianu, Iulia Andraș, Tudor-Eliade Ciuleanu, Nicolae Crișan, Camelia Alexandra Coadă

**Affiliations:** 1 10th Department—Oncology, “Iuliu Haţieganu” University of Medicine and Pharmacy, 400012 Cluj-Napoca, Romania; dulf.daniel@elearn.umfcluj.ro (D.-V.D.); tudor_ciuleanu@hotmail.com (T.-E.C.); 2 Clinical Municipal Hospital Cluj-Napoca, 400139 Cluj-Napoca, Romaniadr.iuliaandras@gmail.com (I.A.); 3 “Prof. Dr. Ion Chiricuță” Institute of Oncology, 400015 Cluj-Napoca, Romania; anamaria.lari.burnar@elearn.umfcluj.ro; 4 “Prof. Dr. Nicolae Stăncioiu” Heart Institute, 400370 Cluj-Napoca, Romania; 5 Department of Pathology, “Iuliu Haţieganu” University of Medicine and Pharmacy, 400012 Cluj-Napoca, Romania; 6 Department of Urology, “Iuliu Haţieganu” University of Medicine and Pharmacy, 400012 Cluj-Napoca, Romania; drnicolaecrisan@gmail.com; 7 Department of Morpho-Functional Sciences, “Iuliu Haţieganu” University of Medicine and Pharmacy, 400012 Cluj-Napoca, Romania; coada_camelia_alexandra@elearn.umfcluj.ro

**Keywords:** urothelial carcinoma, survival, prognosis, risk score, outcome, mortality

## Abstract

**Background:** Perioperative factors significantly impact oncologic outcomes after radical cystectomy (RC) for bladder cancer. This study aimed to identify key perioperative predictors for overall (OS) and progression-free survival (PFS) and to develop a prognostic nomogram for the identification of high-risk patients adapted to the clinical routines and standard of care of our country. **Methods:** We retrospectively analyzed 121 patients undergoing RC (2014–2024). Data on patient demographics, comorbidities, tumor pathology, neoadjuvant treatments, extensive intraoperative factors, and postoperative events were assessed using COX models. A prognostic nomogram for 3-year OS was constructed. **Results:** Median follow-up was 44.33 months. Significant predictors for worse OS included lymphovascular invasion (LVI) (HR 2.22), higher T stage (HR 8.75), N+ status (HR 1.10), and intraoperative complications (HR 3.04). Similar predictors were noted for PFS. The developed nomogram incorporated T-, N-stages, sex, grade, intraoperative complications and early (12 months) recurrence, and was able to significantly identify patients with a higher mortality risk (*p* < 0.001) with a C-index of 0.74. **Conclusions:** Our nomogram for mortality prediction of BC patients offers a promising tool for individualized risk stratification. Further studies are required for its external validation.

## 1. Introduction

Bladder cancer remains a significant global health challenge, ranking among the most common malignancies and contributing substantially to cancer-related morbidity and mortality [1]. For patients diagnosed with muscle-invasive bladder cancer or high-risk non-muscle-invasive disease, radical cystectomy with pelvic lymph node dissection represents the cornerstone of curative-intent treatment [2]. Despite advances in surgical techniques and perioperative care, the oncologic outcomes following radical cystectomy exhibit considerable heterogeneity, even among patients with similar clinicopathological characteristics [3,4]. This variability underscores an unmet need for more refined prognostic tools to better stratify patients, guide individualized counseling, and potentially tailor adjuvant treatment strategies.

Traditional prognostic models for bladder cancer heavily rely on well-established pathological factors such as tumor stage (pT), nodal involvement (pN), histological grade, and the presence of lymphovascular invasion (LVI) [4]. While these elements are undeniably crucial, they primarily reflect tumor biology at a specific point in time and may not fully encapsulate the multifaceted influences on a patient’s long-term trajectory. The entire perioperative period, encompassing the patient’s condition before surgery, the specifics and events of the surgical intervention itself, and the early postoperative course, presents a complex interplay of factors that could also significantly impact oncologic outcomes.

Perioperative factors are diverse and can include patient-related aspects (e.g., comorbidities, nutritional status, and smoking), details of neoadjuvant therapy, specifics of the surgical procedure (e.g., operative time, blood loss, extent of dissection, and surgical technique), and the occurrence of postoperative complications or deviations from an optimal recovery path [5]. These factors may influence cancer outcomes through various mechanisms, including modulation of the systemic inflammatory response, impact on immune function, delays in subsequent adjuvant treatments, or by reflecting the overall physiological insult and resilience of the patient [6]. While individual perioperative variables have been investigated, there is a growing recognition of the need for a more comprehensive assessment of their cumulative prognostic impact.

Despite the known benefits of adjuvant treatments for selected high-risk individuals following radical cystectomy, a significant proportion of eligible patients do not receive such therapies for various reasons, including postoperative frailty or delayed identification of true risk [7]. Consequently, there is a critical need for accurate and early risk stratification tools that go beyond traditional pathological staging. A comprehensive prognostic score, integrating multiple perioperative factors as explored in this study, could play a pivotal role in identifying patients at a genuinely high risk of adverse oncologic outcomes, such as poor overall survival. Such identification would enable clinicians to better tailor postoperative management, potentially guiding decisions towards more intensive surveillance or the timely initiation of appropriate systemic therapies for those most likely to benefit, thereby addressing a key area of unmet need in bladder cancer care. In the current literature various attempts have been made to create prognostic scores to better guide medical decisions in these patients [8,9], but none have been validated in our country. Given the high variability of healthcare systems and treatment options across countries, the applicability of these scores may prove to be limited to regions with more advanced healthcare such as those in Western Europe. Thus, there is a need to develop stratification systems tailored to the clinical routines and healthcare standards of Eastern European countries, such as Romania.

Therefore, this study was undertaken to conduct a thorough retrospective analysis of a wide spectrum of perioperative factors in a cohort of patients undergoing radical cystectomy for bladder cancer at a single institution. The primary objectives were to identify specific preoperative, intraoperative, and postoperative variables significantly associated with overall survival (OS) and progression-free survival (PFS), and subsequently, to develop and internally validate a novel prognostic nomogram incorporating these multifaceted predictors to aid in individualized risk assessment.

## 2. Methods

### 2.1. Study Design and Patients Selection

This study was a single-center, retrospective, analytical cohort study, utilizing data from patients diagnosed with bladder cancer who underwent radical cystectomy at Clinical Municipal Hospital Cluj-Napoca, Cluj-Napoca, Romania. Ethical approval for the study protocol was granted by the institutional review board (approval No. 4/2025).

For inclusion in this analysis focusing on perioperative factors, patients were required to meet the following conditions: (1) surgical intervention via radical cystectomy performed at our institution; (2) a histologically confirmed diagnosis of bladder cancer; (3) availability of comprehensive clinical records; (4) complete survival outcome data; and (5) a detailed pathological report from the cystectomy specimen being on file.

Patients were excluded from the final study cohort if they presented with (1) a documented history of any other primary malignancy, excluding non-melanoma localized skin carcinoma and in situ carcinoma of the cervix; (2) prior systemic chemotherapy or immunotherapy administered for different cancers; (3) previous radiotherapy to the pelvic region; or (4) if crucial data points for the assessment of perioperative factors and outcomes were incomplete or missing.

### 2.2. Data Collection

A retrospective review of medical records was conducted for all patients who underwent radical cystectomy for histologically confirmed bladder cancer with surgical procedures performed between January 2014 and November 2024.

The collected data encompassed several key domains: (1) Patient demographics and baseline clinical information—this included age at the time of surgery, sex, residential background (urban/rural), Body Mass Index (BMI), detailed smoking history (status, current smoking at intervention, number of cigarettes per day, and duration of smoking in years), and overall comorbidity status. (2) Tumor pathological characteristics: histological subtype, tumor grade, pathological T stage and N stage, presence of perineural invasion, and lymphovascular invasion (LVI). (3) Preoperative and neoadjuvant treatment details: variables in this category included the time interval from initial diagnosis to radical cystectomy, administration details of neoadjuvant chemotherapy (NAC) if received (regimens, including number of cycles), and any documented chemotherapy-related toxicities or dose reductions. (4) Intraoperative surgical factors: performance of nerve-sparing techniques, type of urinary diversion, whether the diversion was constructed intracorporeally or extracorporeally, total operative time, and duration of specific surgical steps (cystectomy, lymphadenectomy, and urinary diversion). Intraoperative metrics also included estimated blood loss, the requirement for intraoperative blood transfusions, and the occurrence of any intraoperative complications. These were defined as any unplanned events occurring during surgery that required deviation from the standard procedure, including organ injury, vascular or bowel damage, anesthetic-related issues, or bleeding requiring transfusion. (5) Postoperative management and early recovery: data were collected on the postoperative day of patient mobilization, the day of gastrointestinal transit return, total length of postoperative hospital stay, and the duration for which ureteral stents and urinary catheters were maintained. (6) Postoperative events and complications: the need for preoperative or postoperative blood transfusions, the occurrence and severity of postoperative complications (graded according to the Clavien–Dindo classification and assessed within 30 days of surgery), rates of rehospitalization, and the necessity for surgical reintervention. (7) Oncologic follow-up and survival data: information on tumor recurrence patterns (local, distant, or combined) and event date, vital status and date of death, was obtained following a formal written request to the Civil Registry Office.

Pathological staging of bladder cancer was performed using the American Joint Committee on Cancer (AJCC)/Union for International Cancer Control (UICC) TNM staging system, employing the 7th edition [10] for cases diagnosed up to 2017 and the 8th edition [11] thereafter. The determination of positive lymph node involvement (pN+) was based on histopathological evidence from dissected nodes or on radiologic findings suggestive of metastasis on computed tomography (CT) scans. Assessment for distant metastases (M status) relied on CT imaging of the thorax, abdomen, and pelvis. Histological tumor classification adhered to the World Health Organization (WHO) 2004 guidelines [12], with a progressive shift towards the updated WHO 2016 classification system [13] during the study timeframe. Tumor grade was classified according to WHO 2004/2016 guidelines [12,13], using a two-tiered system (low grade vs. high grade).

### 2.3. Statistical Analysis

R version 4.4.3 (2025-02-28 ucrt)—“Trophy Case” [14] was used for the statistical analysis. Continuous variables were reported using medians and the interquartile range (IQR). Differences between groups were tested with t-tests or Mann–Whitney tests, as appropriate. Categorical variables were reported as absolute frequency and percentages, while significance between groups was tested using Chi-Square tests or Fisher tests as appropriate. OS was defined as the time from patient diagnosis to the time of death or last known alive. Events were defined as death of any cause, while patients still alive at the time of the analysis were censored for the OS analysis. Kaplan–Meier curves were used to represent patients OS while significance was tested using the log-rank test. The reverse Kaplan–Meier method was used to estimate the median follow-up time. Univariable Cox proportional hazard models were created to identify factors associated with PFS and OS. The prediction nomogram was built using the *rms* R package [15]. Candidate variables were selected based on a combination of COX univariable analysis (*p* < 0.1), prior evidence from the literature, and clinical relevance, to ensure a model that is both statistically and clinically sound. Additionally, for the final nomogram, variables were selected using the penalized COX regression using the *glmnet* R package [16]. Internal validation of the nomogram was performed using bootstrap resampling with 1000 iterations. Predictive accuracy was assessed using Harrell’s concordance index (C-index). Decision curve analysis (DCA) was built to evaluate the benefit of the nomogram across various probability thresholds. Hazard ratios (HRs) with 95% confidence intervals (CIs) were reported for each variable. A two-sided *p*-value less than 0.05 was used to define statistical significance.

## 3. Results

### 3.1. General Characteristics

A total of 121 patients were included in this study. Median follow-up time was 44.33 months (IQR 20.03–70.17). The median age was 69 years (IQR 65–73), and the majority were male (79.34%) and urban residents (65.29%). Smoking was a prevalent risk factor, with 74.38% of patients reporting active smoking, a median of 20 cigarettes per day (IQR 20–25), and a smoking history spanning a median of 21 years (IQR 20–30).

Among the cohort, 66.94% had at least one comorbidity, the most common being hypertension (33.88%) and diabetes mellitus (27.27%) (Table 1).

### 3.2. Pathological Features

The predominant histological subtype was urothelial carcinoma, present in 93.39% of cases. Most tumors were high-grade (80%), and the presence of carcinoma in situ (CIS) was noted in 3.67%. Regarding tumor staging, 27.27% of tumors were staged as pT2, followed by pT3 (25.62%) and pT1 (22.31%). Nodal status was negative (pN0) in 71.9%, while positive nodal involvement (pN1–pN3) was observed in 18.18%. Perineural invasion was present in 55.56% of assessed cases (Table 1).

### 3.3. Treatment and Surgical Details

The median time from diagnosis to radical cystectomy was 3 months (IQR 2–5). Lymph node dissection was performed in 88.43% of patients, and nerve-sparing surgery was conducted in 17.02%. Urinary diversion techniques included ileal conduit (47.93%), orthotopic neobladder (43.8%), and continent cutaneous diversion (8.26%). Intracorporeal urinary diversion was performed in 52.89% of cases. NAC was administered to 42.86% of patients, most patients receiving a median of four cycles (IQR 3–4). Chemotherapy-related toxicity was reported in 10.59%, and dose reductions were required in only 3.39% of cases (Table 1). NAC was administered according to prevailing clinical guidelines and available protocols, using either dose-dense MVAC (methotrexate, vinblastine, doxorubicin, and cisplatin) [17] or gemcitabine–cisplatin [18]. Adjuvant chemotherapy (AC), when applied, consisted of gemcitabine–cisplatin or gemcitabine–carboplatin, the latter typically being chosen based on impaired renal function or reduced creatinine clearance.

The median total operative time was 305 min (IQR 200–370), with a median cystectomy duration of 150 min (IQR 130–170), lymphadenectomy time of 50 min (IQR 50–60), and urinary diversion time of 180 min (IQR 160–200). Patients were mobilized by postoperative day 2 (IQR 1–2), and gastrointestinal transit returned by day 3 (IQR 3–5). The median postoperative hospital stay was 8 days (IQR 6–11).

Ureteral stents and urinary catheters were maintained for a median of 27 (IQR 21–33) and 28 days (IQR 23–30), respectively (Table 1).

### 3.4. Complications and Recurrence

The median intraoperative blood loss was 250 mL (IQR 200–400), with intraoperative transfusions required in only 1.65% of cases. Postoperative transfusions were more common (25.62%), and 22.31% of patients required transfusion prior to surgery. Intraoperative complications occurred in 10.74%, while postoperative complications were observed in 9.09%. According to the Clavien–Dindo classification, the majority were grade 2 (53.16%), followed by grade 1 (22.78%) and grade 3 (18.99%). Severe complications (≥grade 3) occurred in 5.06%.

Rehospitalization was required in 37.19% of patients, and surgical reintervention in 3.31%. Tumor recurrence occurred in 35.83% of patients: 9.17% had local recurrence, 21.67% had distant recurrence, and 5% experienced both (Table 1).

### 3.5. Factors Associated with the Progression-Free and Overall Survival of Bladder Cancer Patients

In the COX proportional hazards analysis for OS, several factors were significantly associated with prognosis. Male sex was associated with a decreased risk of death compared to females (HR 0.41, 95% CI: 0.23–0.76, *p* = 0.004). The presence of LVI (HR 2.22, 95% CI: 1.25–3.94, *p* = 0.006), higher T stage (≥T2) (HR 8.75, 95% CI: 2.72–28.22, *p* < 0.001), and positive lymph node status (N+) (HR 1.10, 95% CI: 1.01–1.20, *p* = 0.03) were all associated with significantly worse survival (Table 2).

Regarding surgical variables, pelvic lymph node dissection (HR 0.40, 95% CI: 0.19–0.83, *p* = 0.013), nerve-sparing surgery (HR 0.24, 95% CI: 0.06–0.99, *p* = 0.049), and intracorporeal urinary diversion (HR 0.36, 95% CI: 0.19–0.66, *p* = 0.001) were associated with improved OS. Notably, the use of an ileal conduit (vs. ureterostomy) was associated with better survival (HR 0.30, 95% CI: 0.15–0.60, *p* = 0.001). Longer total surgery duration was slightly but significantly associated with worse OS (HR 1.00, 95% CI: 0.99–1.00, *p* = 0.002). Receiving a blood transfusion intraoperatively was a strong predictor of poor OS (HR 20.75, 95% CI: 4.17–103.39, *p* < 0.001), as were intraoperative complications (HR 3.04, 95% CI: 1.46–6.34, *p* = 0.003). Recurrence within the first year was significantly associated with worse OS (HR 2.00, 95% CI: 1.11–3.60, *p* = 0.022), particularly in cases of distant recurrence (HR 3.54, 95% CI: 1.82–6.89, *p* < 0.001) and combined local and distant recurrence (HR 6.62, 95% CI: 2.41–18.19, *p* < 0.001).

In the PFS analysis, the presence of LVI (HR 1.80, 95% CI: 1.03–3.14, *p* = 0.038) and advanced T stage (≥T2) (HR 5.40, 95% CI: 2.13–13.71, *p* < 0.001) predicted shorter PFS. Nerve-sparing surgery (HR 0.27, 95% CI: 0.08–0.88, *p* = 0.030), use of a conduit urinary diversion (HR 0.42, 95% CI: 0.22–0.77, *p* = 0.005), and intracorporeal diversion (HR 0.43, 95% CI: 0.24–0.77, *p* = 0.005) were significantly associated with longer PFS. Additionally, receiving chemotherapy was associated with improved PFS (HR 0.53, 95% CI: 0.30–0.94, *p* = 0.030), and longer cystectomy duration was modestly associated with increased risk of progression (HR 1.01, 95% CI: 1.00–1.02, *p* = 0.038) (Table 2).

### 3.6. Risk Stratification of Patients Based on Perioperative and Pathological Features

**Figure 1:**
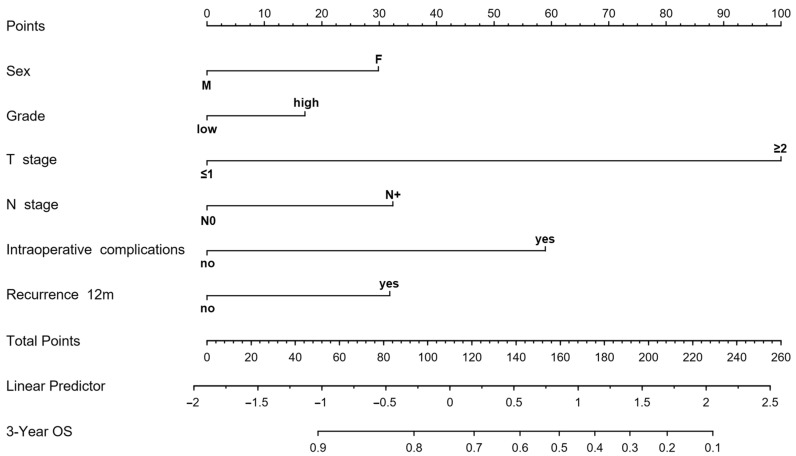
Nomogram predicting the risk of death in patients with bladder cancer, based on sex, tumor grade, pathological T and N stages, intraoperative complications, and recurrence within the first year.

**Figure 2:**
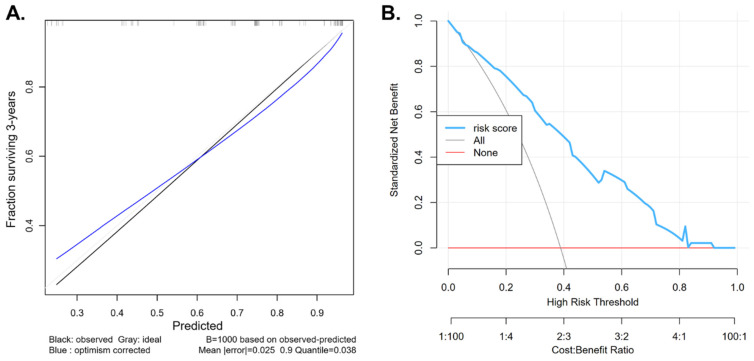
(**A**) Calibration curve of the nomogram showing the balance between the predicted and 3-year survival. The *x*-axis shows the predicted 3-year survival probability while the *y*-axis shows the observed 3-year survival. The gray line represents a perfect calibration while the black line shows the apparent calibration, and the blue line shows the optimism-corrected calibration using bootstrapping. The rug plot represents the frequency distribution of the predicted probabilities. (**B**) Decision Curve Analysis (DCA) of the risk score model. The *x*-axis represents the threshold probability for classifying a patient as high risk, while the *y*-axis shows the standardized net benefit. The blue line indicates the net benefit of using the risk score model. The gray line represents the strategy of treating all patients, and the red line represents treating none.

A prognostic nomogram was developed to estimate 3-year overall survival (OS) in bladder cancer patients, incorporating key clinical and pathological variables including sex, tumor grade, pathological T and N stages, intraoperative complications, and recurrence within the first year (Figure 1). The predictive accuracy as evaluated by the C-index was 0.74, suggesting a good discrimination. On DCA, the nomogram showed a clinical net benefit to predict patient OS in the range of probability of approximately 10–80%, with a good calibration of the model (Figure 2). The results of the internal validation with 1000 iterations showed a slight overfitting with a slope of 0.78, a good calibration as indicated by the small U index (Table 3).

**Figure 3:**
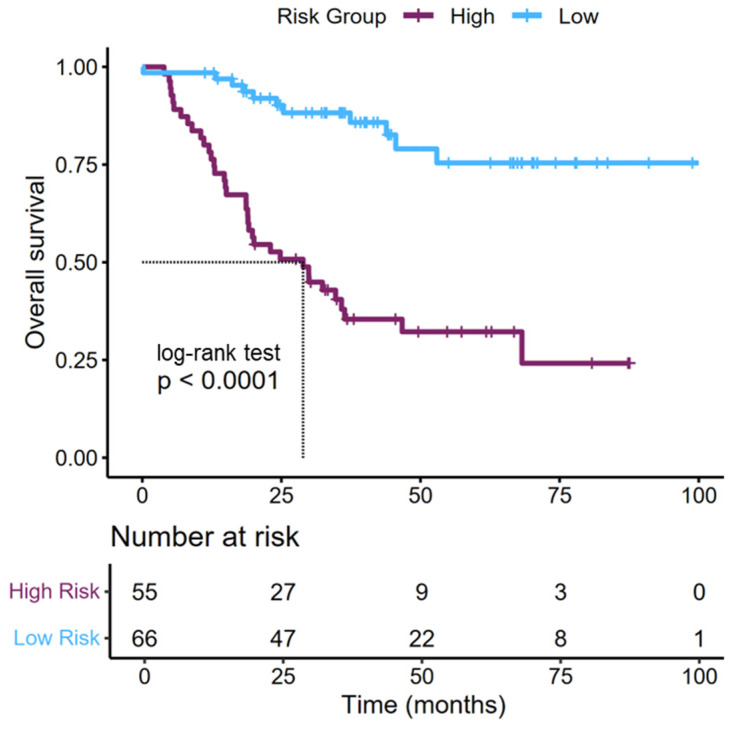
Kaplan–Meier curve showing the overall survival of patients with bladder cancer divided by the risk score derived from the nomogram.

Each variable was assigned a weighted score, and the cumulative score was used to predict the probability of survival at 3-years. Based on the total nomogram-derived score, patients were stratified into low- and high-risk groups for mortality. Kaplan–Meier analysis demonstrated a significant difference in OS between the two risk groups (*p* < 0.001), with low-risk patients having markedly better OS outcomes (Figure 3). In detail, patients with a high-risk score had a median OS of 23 months vs. those with low risk who had a median OS of approximately 68 months (Figure 3).

## 4. Discussion

The present study aimed to identify perioperative predictors of oncologic outcomes in a cohort of 121 patients undergoing radical cystectomy for bladder cancer. Our findings confirm that a multitude of factors including tumor pathology, surgical variables, and postoperative events significantly influence both OS and PFS.

Key pathological features such as the presence of LVI, higher pathological T stage (≥T2), and positive lymph node status (N+) were associated with poorer OS and/or PFS in univariable analysis, consistent with the established literature. Beyond these well-known pathological determinants, our analysis also highlighted the prognostic impact of specific surgical and perioperative management aspects. For instance, performing PLND, nerve-sparing surgery, and the use of intracorporeal urinary diversion were associated with improved survival outcomes. Conversely, factors such as longer total surgery duration, the occurrence of intraoperative complications, and tumor recurrence within the first year emerged as significant predictors of unfavorable overall survival. Furthermore, this study successfully led to the development of a prognostic nomogram incorporating a panel of these diverse perioperative and pathological variables, which effectively stratified patients into distinct risk groups for 3-year OS.

### 4.1. Study Results in the Context of the Published Literature

The pathological characteristics of the resected tumors predictably played a crucial role in determining oncologic outcomes. Our analyses confirmed that advanced pathological T stage (≥pT2) was a powerful predictor of both significantly worse OS and PFS, a finding consistent across virtually all the bladder cancer literature [4]. Similarly, the presence of lymph node metastasis (N+) was significantly associated with poorer OS, underscoring the systemic implications of nodal spread [2]. LVI also emerged as a significant factor for unfavorable OS and PFS in our cohort, reinforcing its role as an indicator of aggressive tumor biology and metastatic potential, as discussed extensively in previous studies [19]. Current European Association of Urology (EAU) guidelines emphasize the importance of a thorough bilateral PLND to optimize staging and oncologic outcomes, recommending an extended template, although the minimum number of lymph nodes that should be retrieved continues to be debated [20]. Multiple studies have demonstrated that a higher lymph node yield during cystectomy correlates with better survival outcomes, underscoring both the diagnostic and therapeutic value of an adequate lymphadenectomy [21,22,23], results which align with our study.

During the study period, updates in the AJCC/UICC TNM staging system (transitioning from the 7th to the 8th edition) and the WHO histological grading system (from the 2004 to the 2016 version) were implemented. These modifications raised the potential for discrepancies in pathological classification over time. However, upon thorough review, we found that the revisions had minimal impact on the core parameters relevant to our cohort. The primary tumor staging (T category) remained unchanged between editions, and grading for muscle-invasive tumors continued to follow the binary low-grade/high-grade system, with no substantive redefinition. All pathology reports were retrospectively reviewed, and a standardized abstraction protocol was used to ensure consistency in staging and grading across the dataset.

While other histopathological features such as high tumor grade and perineural invasion (Pn) are well-recognized markers of aggressive disease in urothelial carcinoma [24] in our analysis for OS and PFS, their prognostic impact was limited possibly due to the relatively small number of patients. However, tumor grade was included in our final prognostic nomogram, suggesting its contribution within a multifactorial predictive model. The prevalence of high-grade disease of our cohort already indicates a generally aggressive tumor profile in patients selected for radical cystectomy.

The type and technique of urinary diversion also appeared to influence prognosis in our cohort. In our univariable analyses, both the use of an ileal conduit (when compared to ureterostomy) and the performance of intracorporeal urinary diversion were associated with improved OS and/or PFS. The choice of urinary diversion is often multifactorial, balancing disease extension, patient preference, comorbidities, and surgeon experience [25]. Such an impact could potentially be mediated through differences in operative stress, length of surgery, early complication rates, speed of postoperative recovery, or variations in the systemic inflammatory response post-surgery [26]. Beyond planned surgical techniques, specific intraoperative complications also demonstrated a profound impact on OS. This highlights that unforeseen adverse events during the surgical procedure can have lasting consequences, possibly by triggering systemic inflammatory responses, delaying recovery, or necessitating further interventions that impact the patient’s overall trajectory [27,28]. Moreover, the presence of complications may be influenced by center-specific practices and surgical expertise, and can reflect the quality of surgical care, an important determinant of patient outcomes.

### 4.2. Nomograms from the Published Literature

A key contribution of this study is the development of a novel prognostic nomogram for predicting 3-year overall survival in patients undergoing radical cystectomy for bladder cancer. This model integrates a comprehensive array of accessible preoperative, intraoperative, pathological, and early postoperative variables, including patient sex, tumor grade, pathological T and N stages, occurrence of intraoperative complications, and tumor recurrence within the first year. Our internal validation demonstrated that the nomogram possesses the ability to discriminate between patients at low versus high risk of mortality, as evidenced by the significant separation of Kaplan–Meier survival curves for the derived risk groups (*p* < 0.001) and a C-index of 0.74. This capacity for risk stratification could have several clinical implications as indicated by the DCA which showed a greater net clinical benefit than either treating all or treating no patients across a broad range of threshold probabilities (10–60%). This range corresponds to typical clinical decision thresholds, indicating that the model could meaningfully inform risk-stratified management. At higher thresholds the model’s benefit diminishes, likely due to reduced sensitivity or increased false positives. Firstly, it can serve as a valuable tool for patient counseling, providing more individualized prognostic information. Secondly, it may aid clinicians in identifying high-risk patients who might benefit most from intensified surveillance strategies or consideration for adjuvant therapies [29].

While clinical factors are readily available and undoubtedly represent valuable tools for predicting patient outcomes, recent advances in complex machine learning models, digital pathology, and the integration of artificial intelligence (AI) have shown promise in improving the prediction of OS and PFS [30,31,32]. Nevertheless, the applicability of these advanced technologies remains limited to a few well-resourced centers, while most hospitals lack the necessary infrastructure.

Recent years have seen the development of multiple prediction scores which consider various features in different combinations [8] out of which some are widely known such as the International Bladder Cancer Nomogram Consortium nomogram for prediction of PFS [9]. The C-indexes of these tools ranged from 0.61 to 0.85 with some showing high variability depending on the country. This is highly relevant as different regions have different standards of care and resources which ultimately impact patients’ outcomes. While robust comparative validation studies are needed, our nomogram offers a unique advantage, as it is, to the best of our knowledge, the only one incorporating early recurrence (within one year post-surgery) into the prediction score. Its limitation is that it becomes applicable only after the first year, not immediately post-surgery. Nevertheless, it can complement existing tools and aid in guiding treatment strategies for recurrent tumors, particularly when a poor prognosis may justify a more aggressive approach.

### 4.3. Future Research Directions

Further research is required for the external validation of our proposed nomogram. Moreover, elucidating the biological mechanisms underlying the observed associations might provide deeper insights into their impact on tumor progression and patient survival [33]. The integration of molecular markers, such as those derived from liquid biopsies or tumor tissue, with the perioperative factors identified in our nomogram, could lead to more refined and personalized prognostic models, potentially identifying patients who would benefit most from novel adjuvant therapies [34]. Finally, exploring the impact of implementing perioperative care pathways or enhanced recovery after surgery (ERAS) protocols, which aim to standardize and optimize many of the factors we analyzed, on the oncologic outcomes of bladder cancer patients undergoing radical cystectomy would be of significant clinical interest [35,36].

### 4.4. Study Limitations and Strengths

The present study possesses several strengths that enhance the validity and relevance of its findings. Firstly, it involves a comprehensive data collection process, encompassing a wide array of demographic, clinical, detailed pathological, specific surgical, intraoperative, and postoperative variables from a reference institution’s experience with radical cystectomy over a 10-year period (2014–2024). This allowed for an in-depth exploration of numerous potential perioperative predictors of oncologic outcome. Secondly, the focus on clinically significant endpoints, namely OS and PFS, provides direct insights into factors affecting long-term patient prognosis. A key output of this research is the development of a novel prognostic nomogram for 3-year OS, which integrates multiple significant perioperative factors and demonstrated good discriminative ability in our cohort. Such a tool has the potential to aid in personalized risk stratification and patient counseling. Furthermore, the median follow-up of 44.33 months was adequate for observing a sufficient number of oncologic events.

Nevertheless, several limitations must be acknowledged. The primary limitation lies in the retrospective design of this study, which can introduce risks of selection bias, information bias due to reliance on pre-existing medical records, and the possibility of unmeasured confounding factors, all of which may have influenced the findings. While we made significant efforts to minimize these biases, such as employing standardized data extraction protocols, cross-checking information from multiple institutional sources, and involving two independent reviewers in data validation, the completeness and consistency of certain variables could not always be fully ensured. Secondly, being a single-center study, the findings, including the performance of the developed nomogram, may have limited external generalizability to other populations or healthcare settings with different patient characteristics or surgical practices. While our cohort size of 121 patients allowed for the identification of several prognostic factors, it may be considered modest for the development of a complex nomogram incorporating numerous variables, potentially increasing the risk of overfitting the model to our specific dataset. Thus, further research is required to validate our model in other clinical settings.

Moreover, during the 10-year study period, updates occurred in the AJCC/UICC TNM staging (7th to 8th edition) and WHO histological grading systems (2004 to 2016). However, these revisions had limited clinical impact in our cohort. Pathological staging and grading were reviewed and harmonized using a standardized protocol, and no major discrepancies were identified that would influence the results.

Furthermore, while data on NAC and AC administration were collected, detailed stratification by specific regimens was not performed since this was not the primary focus of this analysis. We acknowledge this as a limitation and note that variability in systemic therapy may represent a source of unmeasured confounding.

## 5. Conclusions

This study identified a wide spectrum of readily available perioperative factors, comprising patient baseline characteristics, pathological features, specific surgical techniques, intraoperative events, and early postoperative occurrences, that significantly predict oncologic outcomes in patients undergoing radical cystectomy for bladder cancer patients. We developed a novel prognostic nomogram, integrating these factors, which demonstrated a strong ability to stratify patients into distinct mortality risk groups. However, given the modest sample size, these findings should be interpreted with caution and require further validation in larger, independent cohorts before being generalized. Nonetheless, this approach holds significant potential for optimizing follow-up and treatment strategies for bladder cancer patients.

## Figures and Tables

**Table 1:** Characteristics of bladder cancer patients included in this study. n: number of cases; IQR: interquartile range; NAC: neoadjuvant chemotherapy; Pn: perineural invasion.

Variable		Summary Statistics n = 121
**Demographic Characteristics and Risk Factors**		
Age (years), median (IQR)		69 (65;73)
Sex, n (%)	F	25 (20.66)
	M	96 (79.34)
BMI, median (IQR)		26.42 (22.8;29.1)
Area of Residence, n (%)	rural	42 (34.71)
	urban	79 (65.29)
Smoker Status, n (%)	no	31 (25.62)
	yes	90 (74.38)
Number of Cigarettes, median (IQR)		20 (20;25)
Number of Years Smoking, median (IQR)		21 (20;30)
Comorbidities, n (%)	no	40 (33.06)
	yes	81 (66.94)
Blood Hypertension, n (%)	no	80 (66.12)
	yes	41 (33.88)
Diabetes Mellitus, n (%)	no	88 (72.73)
	yes	33 (27.27)
**Pathological Characteristics**		
Histotype, n (%)	urothelial	113 (93.39)
	squamous	4 (3.31)
	neuroendocrine	2 (1.65)
	other	2 (1.65)
Grade, n (%)	low	23 (20)
	high	92 (80)
T, n (%)	T0	12 (9.92)
	T1	27 (22.31)
	T2	33 (27.27)
	T3	31 (25.62)
	T4	18 (14.88)
N, n (%)	N0	87 (71.9)
	N1	10 (8.26)
	N2	11 (9.09)
	N3	1 (0.83)
	Nx	12 (9.92)
Pn, n (%)	absent	12 (44.44)
	present	15 (55.56)
**Surgical Procedures and Treatment**		
Time from Diagnosis to Cystectomy (months), median (IQR)		3 (2;5)
Lymph Node Dissection, n (%)	no	14 (11.57)
	yes	107 (88.43)
Nerve Sparing, n (%)	none	78 (82.98)
	Unilateral	5 (5.32)
	Bilateral	11 (11.7)
Type of Diversion, n (%)	Ureterostomy	58 (47.93)
	Ileal Conduit	53 (43.8)
	Neobladder	10 (8.26)
Intra/Extracorporeal Diversion, n (%)	Intracorporeal	57 (47.11)
	Extracorporeal	64 (52.89)
Treatment, n (%)	NAC	51 (42.86)
	AC	21 (17.65)
	Surgery only	47 (39.5)
Number of NAC Cycles, median (IQR)		4 (3;4)
Toxicity, n (%)	no	76 (89.41)
	yes	9 (10.59)
Need for Dose Reduction, n (%)	no	57 (96.61)
	yes	2 (3.39)
Total Operative Time (minutes), median (IQR)		305 (200;370)
Cystectomy Operative Time (minutes), median (IQR)		150 (130;170)
Lymphadenectomy Operative Time (minutes), median (IQR)		50 (50;60)
Urinary Diversion Operative Time (minutes), median (IQR)		180 (160;200)
Day of Mobilization, median (IQR)		2 (1;2)
Day of First Bowel Movement, median (IQR)		3 (3;5)
Number of Postoperative Hospital Days, median (IQR)		8 (6;11)
Days to Catheter Removal in Patients with Neobladder, median (IQR)		27 (21;33)
Days to Ureteral Stents Removal in Patients with Ileal Conduit, median (IQR)		28 (23;30)
**Complications**		
Estimated Blood Loss (ml), median (IQR)		250 (200;400)
Intraoperative Transfusion, n (%)	no	119 (98.35)
	yes	2 (1.65)
Preoperative Transfusions, n (%)	no	94 (77.69)
	yes	27 (22.31)
Postoperative Transfusions, n (%)	no	90 (74.38)
	yes	31 (25.62)
Intraoperative Complications, n (%)	no	108 (89.26)
	yes	13 (10.74)
Postsurgical Complications, n (%)	no	110 (90.91)
	yes	11 (9.09)
Clavien Classification, n (%)	1	18 (22.78)
	2	42 (53.16)
	3	15 (18.99)
	>3	4 (5.06)
Rehospitalization, n (%)	no	76 (62.81)
	yes	45 (37.19)
Need for Surgical Reintervention, n (%)	no	117 (96.69)
	yes	4 (3.31)
**Follow-up**		
Recurrence Site, n (%)	none	77 (64.16)
	local	11 (9.17)
	distant	26 (21.67)
	local and distant	6 (5)

**Table 2:** COX proportional hazards for OS and PFS. PFS: progression-free survival; OS: overall survival.

		OS		PFS	
Variable		HR (95% CI)	*p*-Value	HR (95% CI)	*p*-Value
**Demographic characteristics and risk factors**					
Age		1.03 (0.99;1.07)	0.140	1.03 (1;1.07)	0.056
Sex, M vs. F		0.41 (0.23;0.76)	0.004	0.6 (0.32;1.12)	0.111
BMI		1 (1;1)	0.136	1 (1;1)	0.415
Smoker status, yes vs. no		0.72 (0.39;1.32)	0.285	1.27 (0.65;2.49)	0.483
Number of cigarettes		1 (0.97;1.04)	0.868	1 (0.97;1.03)	0.942
Number of years smoking		0.99 (0.95;1.03)	0.684	0.98 (0.94;1.02)	0.374
Comorbidities, present vs. absent		0.74 (0.41;1.32)	0.305	0.51 (0.29;0.89)	0.018
Blood hypertension, present vs. absent		0.92 (0.5;1.7)	0.790	1.3 (0.71;2.37)	0.389
Diabetes mellitus, present vs. absent		0.92 (0.48;1.74)	0.788	0.95 (0.5;1.8)	0.880
**Pathological characteristics**					
Grade, high vs. low		2.14 (0.90;5.05)	0.082	1.70 (0.72;4)	0.225
LVI, present vs. absent		2.22 (1.25;3.94)	0.006	1.8 (1.03;3.14)	0.038
T stage, 2+ vs. <2		8.75 (2.72;28.22)	<0.001	5.4 (2.13;13.71)	<0.001
N stage, N+ vs. N0		1.1 (1.01;1.2)	0.030	1.07 (0.99;1.17)	0.099
Incidental prostate cancer, present vs. absent		0.64 (0.29;1.43)	0.273	0.73 (0.35;1.5)	0.388
**Surgical procedures and treatment**					
Lymph node dissection, yes vs. no		0.4 (0.19;0.83)	0.013	0.64 (0.3;1.37)	0.249
Nerve sparing, yes vs. no		0.24 (0.06;0.99)	0.049	0.27 (0.08;0.88)	0.03
Urinary diversion type (ref = ureterostomy)	Ileal Conduit	0.3 (0.15;0.6)	0.001	0.42 (0.22;0.77)	0.005
	Neobladder	0.43 (0.1;1.78)	0.243	0.56 (0.17;1.82)	0.334
Urinary diversion intra/extracorporeal, yes vs. no		0.36 (0.19;0.66)	0.001	0.43 (0.24;0.77)	0.005
Total surgery duration		1 (0.99;1)	0.002	1 (0.99;1)	0.105
Cystectomy duration		1 (0.99;1.01)	0.663	1.01 (1;1.02)	0.038
PLND duration		0.98 (0.96;1)	0.105	1.01 (0.99;1.03)	0.203
Urinary diversion duration		1 (0.98;1.01)	0.655	1.01 (1;1.02)	0.265
Number of days in hospital		0.98 (0.92;1.04)	0.445	1.02 (0.97;1.08)	0.337
Days to Catheter removal (patients with neobladder)		1 (0.95;1.06)	0.983	1.04 (1;1.1)	0.071
Days to Urinary catheter removal (patients with ileal conduit)		1.03 (0.79;1.33)	0.845	0.89 (0.76;1.05)	0.167
Received CHT, yes vs. no		1.15 (0.63;2.09)	0.640	0.53 (0.3;0.94)	0.030
**Complications**					
Quantity of blood lost		1 (1;1)	0.226	1 (1;1)	0.561
Transfusion—during surgery, received vs. no		20.75 (4.17;103.39)	<0.001	3.13 (0.42;23.58)	0.268
Transfusion—after surgery, received vs. no		1.26 (0.64;2.48)	0.499	1.48 (0.8;2.76)	0.211
Number of days to mobilization		1.12 (0.8;1.57)	0.519	0.9 (0.65;1.25)	0.545
Number of days to bowel movement		1.03 (0.8;1.33)	0.811	1.09 (0.87;1.38)	0.445
Intraoperative complications, present vs. absent		3.04 (1.46;6.34)	0.003	1.11 (0.44;2.82)	0.818
Postsurgical complications, present vs. absent		1.79 (0.76;4.23)	0.183	0.9 (0.32;2.51)	0.844
Clavien classification (ref = no complication)	1	0.97 (0.4;2.34)	0.948	1.43 (0.66;3.11)	0.369
	2	1.25 (0.64;2.43)	0.509	1.02 (0.52;2)	0.958
	≥3	0.54 (0.2;1.46)	0.222	0.63 (0.24;1.61)	0.333
Rehospitalization, yes vs. no		1.23 (0.69;2.2)	0.475	1.57 (0.9;2.75)	0.112
Need for surgical reintervention, yes vs. no		1.44 (0.35;5.98)	0.611	0.74 (0.18;3.07)	0.682
**Follow-up**					
Recurrence within the first year, yes vs. no		2.28 (1.29;4.05)	0.005		
Recurrence site (ref = none)	local	2.5 (0.98;6.35)	0.055		
	distant	3.54 (1.82;6.89)	<0.001		
	local and distant	6.62 (2.41;18.19)	<0.001		

**Table 3:** Validation analysis of the nomogram.

Parameter	Original Index	Training	Test	Optimism	Corrected Index
Dxy. Somers’ D rank correlation	0.533	0.551	0.501	0.050	0.483
R2. Explained variation	0.300	0.343	0.268	0.076	0.225
Calibration slope	1.000	1.000	0.777	0.223	0.777
D. Discrimination index	0.098	0.118	0.086	0.032	0.066
U. Unreliability index	−0.005	−0.005	0.015	−0.020	0.015
Q. Quality index	0.103	0.123	0.070	0.052	0.051
g. Goodness-of-fit	1.342	1.636	1.185	0.451	0.891

## Data Availability

Data is available upon reasonable request.

## References

[1] Ferlay J., Ervik M., Lam F., Laversanne M., Colombet M., Mery L., Piñeros M., Znaor A., Soerjomataram I., Bray F. Global Cancer Observatory: Cancer Today. https://gco.iarc.who.int/today/.

[2] van der Heijden A.G., Bruins H.M., Carrion A., Cathomas R., Compérat E., Dimitropoulos K., Efstathiou J.A., Fietkau R., Kailavasan M., Lorch A. (2025). European Association of Urology Guidelines on Muscle-Invasive and Metastatic Bladder Cancer: Summary of the 2025 Guidelines. Eur. Urol..

[3] Minervini A., Di Maida F., Tasso G., Mari A., Bossa R., Sforza S., Grosso A.A., Tellini R., Vittori G., Siena G. (2021). Robot Assisted Radical Cystectomy with Florence Robotic Intracorporeal Neobladder (FloRIN): Analysis of Survival and Functional Outcomes after First 100 Consecutive Patients upon Accomplishment of Phase 3 IDEAL Framework. Eur. J. Surg. Oncol..

[4] Waraich T.A., Khalid S.Y., Ali A., Kathia U.M. (2023). Comparative Outcomes of Radical Cystectomy in Muscle-Invasive Bladder Cancer: A Systematic Review and Meta-Analysis. Cureus.

[5] Zhang L., Wu B., Zha Z., Qu W., Zhao H., Yuan J. (2019). Clinicopathological Factors in Bladder Cancer for Cancer-Specific Survival Outcomes Following Radical Cystectomy: A Systematic Review and Meta-Analysis. BMC Cancer.

[6] Lopez-Beltran A., Cookson M.S., Guercio B.J., Cheng L. (2024). Advances in Diagnosis and Treatment of Bladder Cancer. BMJ.

[7] Esteban-Villarrubia J., Torres-Jiménez J., Bueno-Bravo C., García-Mondaray R., Subiela J.D., Gajate P. (2023). Current and Future Landscape of Perioperative Treatment for Muscle-Invasive Bladder Cancer. Cancers.

[8] Sarrió-Sanz P., Martinez-Cayuelas L., Lumbreras B., Sánchez-Caballero L., Palazón-Bru A., Gil-Guillén V.F., Gómez-Pérez L. (2022). Mortality Prediction Models after Radical Cystectomy for Bladder Tumour: A Systematic Review and Critical Appraisal. Eur. J. Clin. Investig..

[9] Bochner B.H., Kattan M.W., Vora K.C., International Bladder Cancer Nomogram Consortium (2006). Postoperative Nomogram Predicting Risk of Recurrence After Radical Cystectomy for Bladder Cancer. J. Clin. Oncol..

[10] Edge S.B., American Joint Committee on Cancer (2010). AJCC Cancer Staging Handbook: From the AJCC Cancer Staging Manual.

[11] Amin M.B., Greene F.L., Edge S.B., American Joint Committee on Cancer (2017). AJCC Cancer Staging Manual.

[12] Eble J.N., Sauter G., Epstein J., Sesterhenn I. (2004). Pathology and Genetics of Tumours of the Urinary System and Male Genital Organs.

[13] Moch H., Humphrey P., Ulbright T., Reuter V. (2016). WHO Classification of Tumours of the Urinary System and Male Genital Organs.

[14] R Core Team (2021). R: A Language and Environment for Statistical Computing.

[15] Harrell F.E. (2025). Rms: Regression Modeling Strategies. R Package Version 8.0. https://cran.r-project.org/web/packages/rms/index.html.

[16] Tay J.K., Narasimhan B., Hastie T. (2023). Elastic Net Regularization Paths for All Generalized Linear Models. J. Stat. Softw..

[17] Choueiri T.K., Jacobus S., Bellmunt J., Qu A., Appleman L.J., Tretter C., Bubley G.J., Stack E.C., Signoretti S., Walsh M. (2014). Neoadjuvant Dose-Dense Methotrexate, Vinblastine, Doxorubicin, and Cisplatin With Pegfilgrastim Support in Muscle-Invasive Urothelial Cancer: Pathologic, Radiologic, and Biomarker Correlates. J. Clin. Oncol..

[18] Adamo V., Magno C., Spitaleri G., Garipoli C., Maisano C., Alafaci E., Adamo B., Rossello R., Scandurra G., Scimone A. (2005). Phase II Study of Gemcitabine and Cisplatin in Patients with Advanced or Metastatic Bladder Cancer: Long-Term Follow-up of a 3-Week Regimen. Oncology.

[19] Mari A., Kimura S., Foerster B., Abufaraj M., D’Andrea D., Gust K.M., Shariat S.F. (2018). A Systematic Review and Meta-Analysis of Lymphovascular Invasion in Patients Treated with Radical Cystectomy for Bladder Cancer. Urol. Oncol. Semin. Orig. Investig..

[20] Nakagawa T. (2021). Lymph Node Dissection for Bladder Cancer: Current Standards and the Latest Evidence. Int. J. Urol..

[21] Koppie T.M., Vickers A.J., Vora K., Dalbagni G., Bochner B.H. (2006). Standardization of Pelvic Lymphadenectomy Performed at Radical Cystectomy: Can We Establish a Minimum Number of Lymph Nodes That Should Be Removed?. Cancer.

[22] Packiam V.T., Tsivian M., Boorjian S.A. (2020). The Evolving Role of Lymphadenectomy for Bladder Cancer: Why, When, and How. Transl. Androl. Urol..

[23] Jena R., Shrivastava N., Sharma A.P., Choudhary G.R., Srivastava A. (2021). The Adequacy of Pelvic Lymphadenectomy During Radical Cystectomy for Carcinoma Urinary Bladder: A Narrative Review of Literature. Front. Surg..

[24] Leissner J., Koeppen C., Wolf H.K. (2003). Prognostic Significance of Vascular and Perineural Invasion in Urothelial Bladder Cancer Treated with Radical Cystectomy. J. Urol..

[25] Lee R.K., Abol-Enein H., Artibani W., Bochner B., Dalbagni G., Daneshmand S., Fradet Y., Hautmann R.E., Lee C.T., Lerner S.P. (2014). Urinary Diversion after Radical Cystectomy for Bladder Cancer: Options, Patient Selection, and Outcomes. BJU Int..

[26] An S., Shi L., Liu Y., Ren L., Zhang K., Zhu M. (2024). Comparison of Extracorporeal and Intracorporeal Urinary Diversion after Robot-Assisted Radical Cystectomy for Bladder Cancer: A Meta-Analysis. Am. J. Mens. Health.

[27] Razdan S., Sljivich M., Pfail J., Wiklund P.K., Sfakianos J.P., Waingankar N. (2021). Predicting Morbidity and Mortality after Radical Cystectomy Using Risk Calculators: A Comprehensive Review of the Literature. Urol. Oncol..

[28] Zhou Y., Li R., Liu Z., Qi W., Lv G., Zhong M., Liu X., Zhu M., Jiang Z., Chen S. (2023). The Effect of the Enhanced Recovery after Surgery Program on Radical Cystectomy: A Meta-Analysis and Systematic Review. Front. Surg..

[29] Shariat S.F., Karakiewicz P.I., Godoy G., Lerner S.P. (2009). Use of Nomograms for Predictions of Outcome in Patients with Advanced Bladder Cancer. Ther. Adv. Urol..

[30] He Q., Xiao B., Tan Y., Wang J., Tan H., Peng C., Liang B., Cao Y., Xiao M. (2024). Integrated Multicenter Deep Learning System for Prognostic Prediction in Bladder Cancer. npj Precis. Oncol..

[31] Bai Z., Osman M., Brendel M., Tangen C.M., Flaig T.W., Thompson I.M., Plets M., Scott Lucia M., Theodorescu D., Gustafson D. (2025). Predicting Response to Neoadjuvant Chemotherapy in Muscle-Invasive Bladder Cancer via Interpretable Multimodal Deep Learning. npj Digit. Med..

[32] Song Q., Seigne J.D., Schned A.R., Kelsey K.T., Karagas M.R., Hassanpour S. (2020). A Machine Learning Approach for Long-Term Prognosis of Bladder Cancer Based on Clinical and Molecular Features. AMIA Jt. Summits Transl. Sci. Proc..

[33] Tu Y., Wang S., Wang H., Zhang P., Wang M., Liu C., Yang C., Jiang R. (2024). The Role of Perioperative Factors in the Prognosis of Cancer Patients: A Coin Has Two Sides. J. Biomed. Res..

[34] Soria F., Krabbe L.-M., Todenhöfer T., Dobruch J., Mitra A.P., Inman B.A., Gust K.M., Lotan Y., Shariat S.F. (2019). Molecular Markers in Bladder Cancer. World J. Urol..

[35] Ma R., Sheybaee Moghaddam F., Ghoreifi A., Ladi-Seyedian S., Cai J., Miranda G., Aron M., Schuckman A., Desai M., Gill I. (2024). The Effect of Enhanced Recovery after Surgery on Oncologic Outcome Following Radical Cystectomy for Urothelial Bladder Carcinoma. Surg. Oncol..

[36] Sung L.H., Yuk H.D. (2020). Enhanced Recovery after Surgery of Patients Undergoing Radical Cystectomy for Bladder Cancer. Transl. Androl. Urol..

